# 3D Printing of Polysaccharide-Based Self-Healing Hydrogel Reinforced with Alginate for Secondary Cross-Linking

**DOI:** 10.3390/biomedicines9091224

**Published:** 2021-09-15

**Authors:** Hyun-Ho Roh, Hyun-Seung Kim, Chunggoo Kim, Kuen-Yong Lee

**Affiliations:** 1 Department of Bioengineering, Hanyang University, Seoul 04763, Korea; yes9268@naver.com (H.-H.R.); hyuns9393@naver.com (H.-S.K.); cung4060@nate.com (C.K.); 2 Institute of Nano Science and Technology, Hanyang University, Seoul 04763, Korea

**Keywords:** 3D printing, self-healing hydrogel, secondary cross-linking, polysaccharide, tissue engineering

## Abstract

Three-dimensional (3D) bioprinting has been attractive for tissue and organ regeneration with the possibility of constructing biologically functional structures useful in many biomedical applications. Autonomous healing of hydrogels composed of oxidized hyaluronate (OHA), glycol chitosan (GC), and adipic acid dihydrazide (ADH) was achieved after damage. Interestingly, the addition of alginate (ALG) to the OHA/GC/ADH self-healing hydrogels was useful for the dual cross-linking system, which enhanced the structural stability of the gels without the loss of their self-healing capability. Various characteristics of OHA/GC/ADH/ALG hydrogels, including viscoelastic properties, cytotoxicity, and 3D printability, were investigated. Additionally, potential applications of 3D bioprinting of OHA/GC/ADH/ALG hydrogels for cartilage regeneration were investigated in vitro. This hydrogel system may have potential for bioprinting of a custom-made scaffold in various tissue engineering applications.

## 1. Introduction

Three-dimensional (3D) printing technology has been widely used to fabricate various artificial tissues and organs with tissue engineering approaches [1,2,3]. It can offer improved versatility by delivering accurate control over the spatial distributions of cells as well as biomaterials [4]. The 3D printing technology enables the production of complex tissues with high-resolution precision [5]. The use of biocompatible materials can also lead to biomimetic microenvironments [6]. Hyaluronate (HA) is a naturally occurring polymer and is the main component of the extracellular matrix (ECM) of native cartilage [7,8]. HA can interact with chondrocyte surfaces and has been widely proposed as a suitable material for cartilage regeneration [9,10,11,12]. Alginate (ALG) is a potential biomaterial that can be added to improve the inherently unfavorable mechanical properties of HA-based materials in the presence of calcium ions [13].

Hydrogels have been proposed as potential 3D bioinks because of their cell-friendly characteristics [14,15]. They can provide oxygen, nutrients, and growth factors for cells [16]. They can also act as ECM, which can collect cells and fabricate tissue structures [16,17,18]. Hydrogels are often used in extrusion-based bioprinting [19,20], a field in which significant progress has been made over the last several years [4]. Mechanical dispenser systems are used in extrusion-based bioprinting for depositing bioinks in the form of filaments [1]. They have been successfully used to engineer various tissue constructs, including cartilage, bone, and skin [2]. However, several problems, including the production of shear stress during the extrusion processes, have been obstacles to the printing of hydrogels [21,22]. Shear stress can break hydrogel structures, causing a printed structure fracture [23,24]. It can also damage cells, leading to cell death [25]. Therefore, extrusion-based bioprinting requires bioinks that have shear thinning properties in order to overcome these obstacles [26].

Self-healing materials can restore their structures and functions after breakage [27,28,29,30]. Self-healing hydrogels can be used for extrusion-based printing because of their ability to autonomously heal and recover their mechanical integrity after extrusion through the nozzle [31,32]. The gel–fluid transition caused by shear stress and rapid self-healing ability of hydrogels is an important factor when applying it to 3D printing [33,34,35,36].

**Figure 1:**
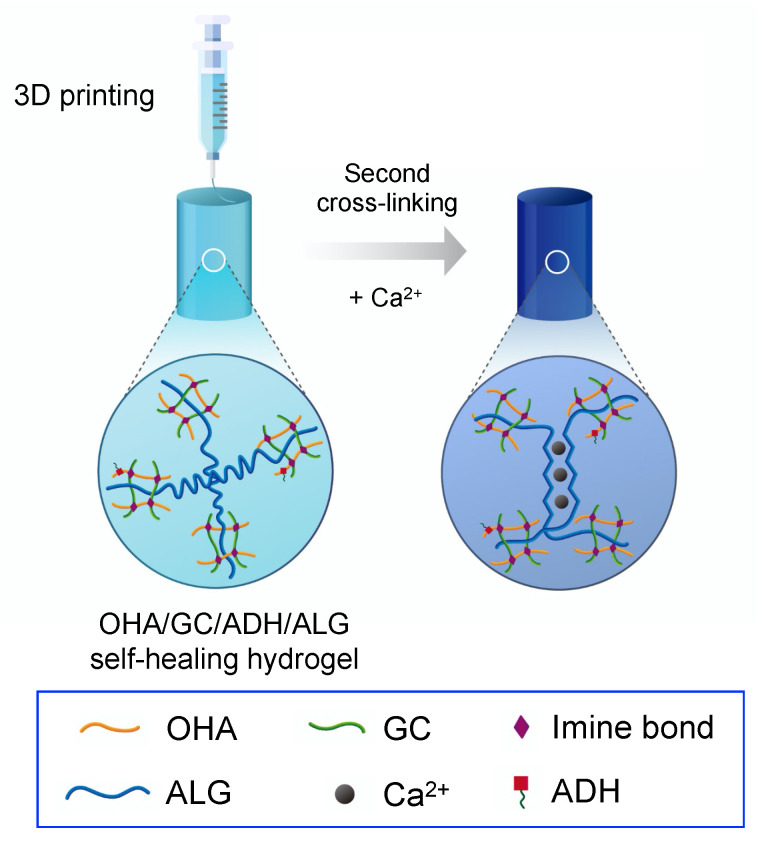
Schematic illustration of fabrication of dual cross-linked 3D construct using an extrusion printing method. Self-healing hydrogel was prepared from oxidized hyaluronate (OHA), glycol chitosan (GC), adipic acid dihydrazide (ADH), and alginate (ALG) by a covalent cross-linking reaction. Second cross-linking was carried out in the presence of calcium ions after the printing process.

In this study, hydrogels with self-healing abilities were proposed to overcome these limitations. The aldehyde group of oxidized hyaluronate (OHA) reacted with the amine group of glycol chitosan (GC) to form a Schiff base, thereby forming a hydrogel. The addition of adipic acid dihydrazide (ADH) induced competition between the imine bond (OHA/GC) and the acylhydrazone bond (OHA/ADH), resulting in chemical self-healing. In addition, secondary cross-linking using ALG and calcium ions was confirmed to enhance the mechanical properties and structural stability of OHA/GC/ADH self-healing hydrogels (Figure 1). It was hypothesized that a dual cross-linking system (e.g., covalent cross-linking and ionic cross-linking) could allow for the fabrication of 3D constructs with well-defined structures, prolonged persistence, and reinforced stiffness, compared with conventional covalent self-healing hydrogel systems (e.g., OHA/GC/ADH hydrogel). Self-healing OHA/GC/ADH/ALG hydrogels were fabricated without using any chemical cross-linkers. The self-healing capabilities of the gels were confirmed before starting the second cross-linking process. Chondrogenic differentiation of cells encapsulated in OHA/GC/ADH/ALG hydrogels was also investigated in vitro.

## 2. Materials and Methods

### 2.1. Materials

Sodium hyaluronate (Mw = 2500 kDa) was purchased from Lifecore (Chaska, MN, USA). Sodium hyaluronate with a molecular weight of 1000 kDa was supplied by Humedix (Anyang, Korea). Sodium alginate (Mw = 250 kDa) was purchased from FMC Biopolymer (Sandvika, Norway). GC (Mw = 50 kDa) was provided by Wako (Osaka, Japan), and 1-Ethyl-3-(dimethylaminopropyl) carbodiimide (EDC) was purchased from Proteochem (Hurricane, UT, USA). *N*-Hydroxysulfosuccinimide sodium salt (sulfo-NHS) was purchased from Covachem (Loves Park, IL, USA). Adipic acid dihydrazide, sodium periodate, 2-(*N*-morpholino)ethanesulfonic acid (MES) hydrate, calcium chloride, human transferrin, and activated charcoal were obtained from Sigma Aldrich (St. Louis, MO, USA). Dulbecco’s phosphate-buffered saline (DPBS), fetal bovine serum (FBS), Dulbecco’s modified Eagle’s medium nutrient mixture F-12 (DMEM/F-12), and penicillin-streptomycin (PS) were supplied by Gibco (Grand Island, NY, USA). A live/dead viability/cytotoxicity kit was purchased from Invitrogen (Waltham, MA, USA).

### 2.2. Preparation of Oxidized Hyaluronate and Glycol Chitosan

HA (1 g) was dissolved in 90 mL of distilled water overnight. Sodium periodate (0.2673 g) was dissolved in 10 mL of distilled water, followed by the addition to the HA solution without light. The mixture was left to react for 24 h to allow for oxidation. The solution was then purified by dialysis against deionized water with sodium chloride for 4 days. The solution was then treated with charcoal and filtered (0.22-µm pore size). The solution (100 mL) was frozen in a deep freezer at −80 °C for 24 h and then lyophilized for 4 days (FreeZone 6 Liter Benchtop Freeze Dry System, Labconco; Kansas City, MO, USA). GC (1 g) was also dissolved in distilled water, and the same process as described above was used to prepare this solution.

### 2.3. Hydrogel Fabrication and Characterization

OHA (2 wt%) and ALG (0.3 wt%) were dissolved in DPBS overnight and GC (1 wt%) and ADH (0.3 wt%) were also dissolved in DPBS overnight. Then, the OHA/ALG solution was mixed with the GC/ADH solution to induce the formation of a hydrogel. Fourier transform infrared spectroscopy (Nicolet IS50, Thermo; Waltham, MA, USA) was used to confirm the formation of the aldehyde groups of OHA and the presence of imine and acylhydrazone bonds in the hydrogel. The viscoelastic properties of the hydrogels were measured using a rotational rheometer (Bohlin Gemini 150, Malvern; Worcestershire, UK) equipped with a cone-and-plate fixture (20 mm diameter plate, 4° cone angle) at 37 °C.

### 2.4. Confirmation of Self-Healing Property of the Hydrogel

A rotational rheometer (Bohlin Gemini 150, Malvern; Worcestershire, UK) was used to test the self-healing properties of the OHA/GC/ADH/ALG hydrogels ([OHA] = 2 wt%, [GC] = 1 wt%, [ADH] = 0.3 wt%, [ALG] = 0.3 wt%). The oscillatory strain was repeatedly altered from 1% to 300% for an evaluation of the self-healing behavior.

### 2.5. Three-Dimensional Printing

A three-dimensional (3D) printer (Invivo, Rokit; Seoul, Korea) was used to fabricate various 3D constructs using an extrusion method. A disposable cartridge was used for printing OHA/GC/ADH/ALG hydrogels. Motor pressure was 300 N and a 25-guage needle was used as a nozzle. Printing speed and temperature were kept at 8 mm/s and 25 °C, respectively. Tinkercad^®^ was used for modeling the 3D constructs (Autodesk; San Rafael, CA, USA). After 3D printing, calcium chloride was added to the printed construct for the formation of ionic cross-linking between ALG and the calcium ions ([Ca^2+^] = 60 mM). Then, any residual calcium ions were removed by washing the product with DPBS 3 times.

### 2.6. Cell Culture

The ATDC5 cell line was purchased from RIKEN Cell Bank (Tsukuba, Japan) and cells were cultured in a DMEM/F-12 growth medium containing 5% FBS, 1% PS, 10 μg/mL human transferrin, and 3 × 10^−8^ M sodium selenite. Bovine insulin (10 μg/mL) was added to the medium for chondrogenic differentiation. Three-dimensional cultures of ATDC5 cells were carried out in a spinner flask. OHA/GC/ADH/ALG hydrogels encapsulating ATDC5 cells (1 × 10^7^ cells/mL) were prepared in the shape of disks. Then, the cell-encapsulated gel disks were incubated at 37 °C.

### 2.7. In Vitro Cell Viability

ATDC5 cells were seeded in 96-well tissue culture plates ([cell] = 1 × 10^5^ cells per well) for an analysis of the cytotoxicity of the individual hydrogel components. Cells were treated with each component (OHA, GC, ADH, ALG; [polymer] = 500–2000 μg/mL) solution and 10 μL of EZ-cytox was added to each well and incubated for 4 h. The cell viability was then evaluated.

The live/dead assay was performed over 4 h according to the manufacturer’s instructions using ATDC5 cells encapsulated in OHA/GC/ADH/ALG hydrogel disks (1 × 10^7^ cells/mL). Reagent EthD-1 and calcein AM were added to the hydrogel disks. After 30 min of incubation, images were taken using a fluorescence microscope (ECLIPSE TE2000-E, Nikon; Japan). The number of live and dead cells was counted in the images for an evaluation of cell viability.

### 2.8. Quantification of Gene Expression

ATDC5 cells in gel disks (diameter = 10 mm, thickness = 1 mm) were cultured for 3 weeks using media containing bovine insulin (10 μg/mL), and chondrogenic differentiation was evaluated by a reverse transcription polymerase chain reaction (RT-PCR) and real-time PCR analyses.

The cells retrieved from the gel disks were treated with RNAiso Plus reagent (Takara, Japan) for RNA extraction. The expression of chondrogenic marker genes, including *SOX-9* and *collagen type II* (*COL-2*), was evaluated using an RT-PCR thermal cycler machine (Takara). The sequences of the primers were as follows: *GAPDH*, 5′-CCATCACCATCTTCCAGGAGCGA-3′, 5′-GGATGACCTTGCCCACAGCCTTG-3′ (447 bp); *SOX-9*, 5′-ATCGGTGAACTGA-GCAGCGAC-3′, 5′-GCCTGCTGCTTCGACATCCA-3′ (200 bp); *COL-2*, 5′-AAGAGCGGTGACTACTGGATAG-3′, 5′-TGCTGTCTCCATAGCTGAAGT-3′ (214 bp). The gene expression level was also quantified by comparison with that of *β-actin*, the reference gene cycle threshold, using an ABI PRISM 7500 Real-Time PCR system (Applied Biosystems, Waltham, MA, USA) and SensiMax SYBR (Bioline, Memphis, TN, USA). The sequences of the primers were as follows: *β-actin*, 5′-CCCTGAA-CCCTAAGGCCAAC-3′; 5′-GCATACAGGGACAGCACAGC-3′; *SOX-9*, 5′-AAGTCGGAGAGCCGAGAGCG-3′, 5′-ACGAAACCGGGGCCACTTGC-3′; *COL-2*, 5’-CACACTGGTAAGTGGGGCAAGACCG-3′, 5-GGATTGTGTTGTTTCAGGGTTCGGG-3′.

### 2.9. Statistical Analysis

All data are presented as mean ± standard deviation (*n* = 4).

## 3. Results and Discussion

### 3.1. Preparation and Characterization of the OHA/GC/ADH/ALG Hydrogel

OHA/ALG and GC/ADH solutions were mixed for the preparation of hydrogels. Hydrogel formation was investigated using FT-IR (Figure S1). A peak denoting an aldehyde group (1720 cm^−1^) appeared after the oxidation reaction of HA, and an imine bond (1456 cm^−1^) appeared when GC was added. Peaks at 3291 cm^−1^ and 3323 cm^−1^, which are derived from the N-H symmetric and asymmetric stretching vibrations of acylhydrazone bonds, were also observed.

The effects of alginate on the characteristics of the OHA/GC/ADH gels are listed in Table 1. First, the concentration of alginate was varied between 0.3 wt% and 1.2 wt%, and the optimum concentration was determined based on the values of the storage shear modulus (G’) and gelation time. The mechanical stiffness decreased as the alginate content increased. Secondary cross-linking improved the mechanical properties of the hydrogel, but an increase in alginate concentration reduced the hydrogel stiffness by more than 0.6 wt%. Therefore, the amount of alginate in OHA/GC/ADH gel was fixed at 0.3 wt% for further studies.

**Figure 2:**
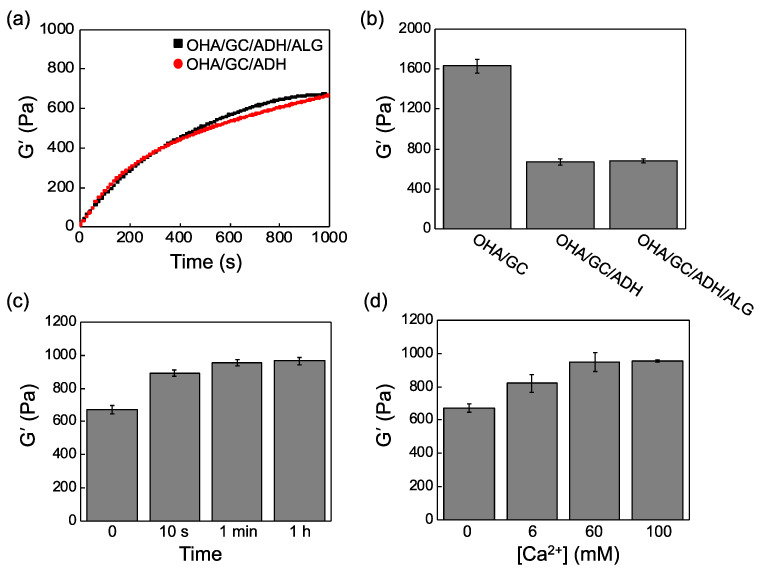
(**a**) Gelation kinetics of OHA/GC/ADH hydrogels prepared in the presence of ALG (black squares) and in the absence of ALG (red circles). (**b**) Storage shear moduli of OHA/GC, OHA/GC/ADH, and OHA/GC/ADH/ALG hydrogels ([OHA] = 2 wt%, [GC] = 1 wt%, [ADH] = 0.3 wt%, [ALG] = 0.3 wt%). Storage shear moduli of OHA/GC/ADH/ALG hydrogels prepared with various (**c**) second cross-linking times ([Ca^2+^] = 60 mM) and (**d**) calcium chloride concentrations (cross-linking time = 1 min).

Next, the gelation kinetics were investigated (Figure 2a). Complete gelation time was determined to be when the storage shear modulus reached a constant value. Gelation generally occurs within a few seconds, but complete gelation occurs only after approximately 10 min. OHA/GC/ADH/ALG hydrogels ([OHA] = 2 wt%, [GC] = 1 wt%, [ADH] = 0.3 wt%, [ALG] = 0.3 wt%) were finally chosen and used for further experiments. The storage shear moduli of the OHA/GC gel, the OHA/GC/ADH gel, and the OHA/GC/ADH/ALG gel were 1630, 670, and 680 Pa, respectively. OHA/GC/ADH and OHA/GC/ADH/ALG hydrogels exhibited similar storage shear moduli (Figure 2b).

A calcium chloride solution was added to generate a second cross-linking between ALG and the calcium ions for enhancing mechanical stiffness and structural stability. The effects of the exposure time and concentration of calcium chloride on the stiffness of the OHA/GC/ADH/ALG gel were investigated (Figure 2c,d). The storage shear modulus of the OHA/GC/ADH/ALG hydrogel increased with increasing calcium chloride concentration and cross-linking time. The optimum calcium chloride concentration and cross-linking time were determined by measuring the mechanical stiffness and viscosity of the second cross-linked hydrogel.

**Figure 3:**
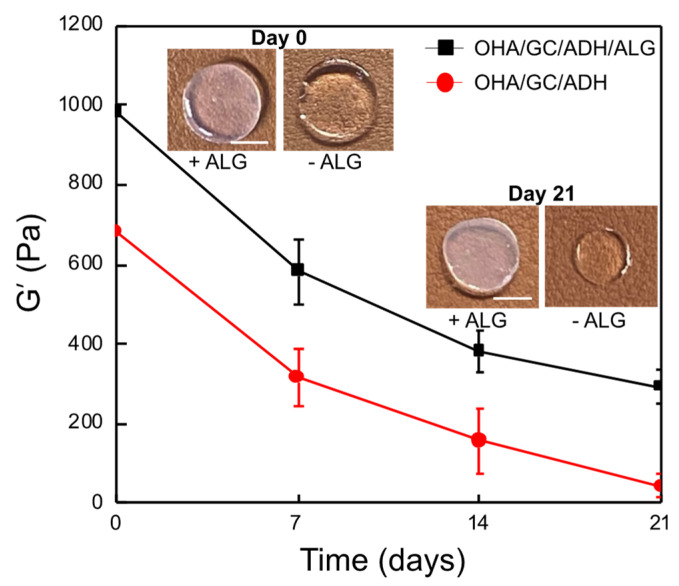
Changes in storage shear moduli of OHA/GC/ADH/ALG hydrogels (black squares) and OHA/GC/ADH hydrogels (red circles) ([OHA] = 2 wt%, [GC] = 1 wt%, [ADH] = 0.3 wt%, [ALG] = 0.3 wt%, [Ca^2+^] = 60 mM). All gel disks (10 mm diameter, 1 mm thick) were prepared, treated with calcium ions for second cross-linking ([Ca^2+^] = 60 mM, 1 min), and incubated in DPBS at 37 °C for 3 weeks (scale bar, 0.5 mm).

The effects of the use of alginate as a secondary cross-linking molecule on the storage modulus and structural stability were next analyzed. A substantial decrease in G′ of the OHA/GC/ADH gel was observed after 3 weeks of incubation in DPBS at 37 °C. However, the secondary cross-linked OHA/GC/ADH/ALG gel maintained more than 40% of its G′ value after 3 weeks of incubation (Figure 3). These results clearly indicate that secondary cross-linking of OHA/GC/ADH/ALG gels can improve the stability of the OHA/GC/ADH gel over a prolonged time period.

### 3.2. Self-Healing Properties of OHA/GC/ADH/ALG Hydrogels

**Figure 4:**
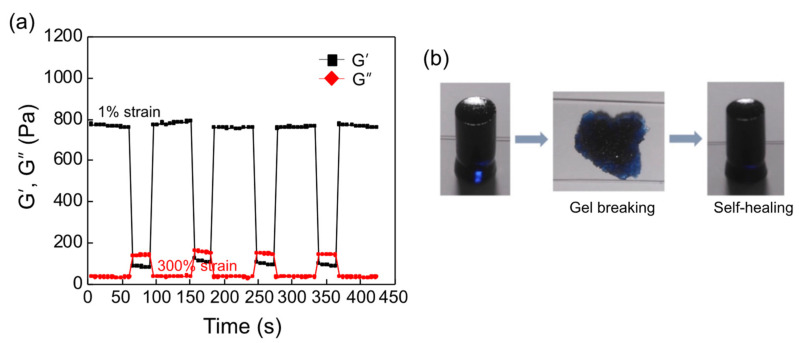
(**a**) The repeated self-healing behavior of OHA/GC/ADH/ALG hydrogels was confirmed by measuring the viscoelastic properties at 300% strain ([OHA] = 2 wt%, [GC] = 1 wt%, [ADH] = 0.3 wt%, [ALG] = 0.3 wt%). (**b**) Macroscopic observation of self-healing behavior of the gel using a cylindrical-shaped mold (5 mm diameter, 10 mm height). The gel was broken and placed into the mold, and mostly recovered its original shape within 10 min.

A rotational viscometer was utilized to confirm the self-healing behavior of the OHA/GC/ADH/ALG hydrogels. The hydrogel was fabricated and cross-linked through alginate-calcium cross-linking. Next, the storage shear modulus of the hydrogel was measured, and a high strain was applied in order to break the gel structure. When an excess strain (300%) was imposed, the loss shear modulus (G’’) was higher than the storage shear modulus (G’), indicating that the hydrogel structure was completely crushed and the gel flow was observed (Figure 4a). Then, the original 1% strain was applied to the crushed hydrogel. The storage shear modulus of the OHA/GC/ADH/ALG hydrogel immediately recovered to its original value (Figure 4a). The self-healing behavior of the hydrogel could be repeated several times. Macroscopic observation using a cylindrical mold also confirmed the self-healing behavior of the gel (Figure 4b).

### 3.3. Three-Dimensional Bioprinting with Self-Healing Hydrogels

**Figure 5:**
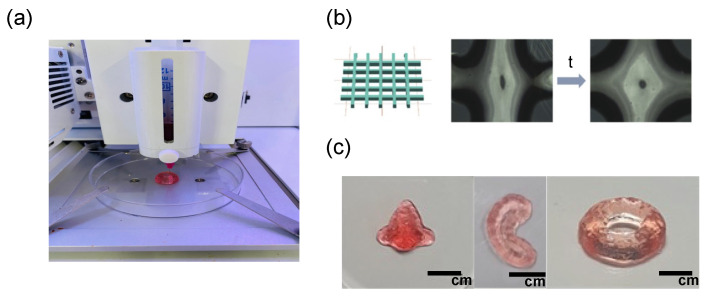
(**a**) Three-dimensional printing of self-healing OHA/GC/ADH/ALG hydrogel encapsulating ATDC5 cells. (**b**) Microscopic images of 3D-printed filaments of OHA/GC/ADH/ALG self-healing hydrogel and (**c**) 3D-printed constructs of various shapes.

OHA/GC/ADH/ALG hydrogels were loaded and printed using disposable cartridges (Figure 5a). A strong shear force was applied to the hydrogel during the printing process, which broke the hydrogel structure. However, self-healing occurred immediately, and the hydrogel maintained its fiber form (Figure 5b). The printed fibers were stacked to form a layer-by-layer laminated structure without collapsing. The printed fibers were finally joined together by self-healing to form a single structure. Then, a calcium chloride solution was added to the printed hydrogels to enhance the mechanical stiffness and structural maintenance. The 3D constructs containing ATDC5 cells of various shapes were printed (Figure 5c).

### 3.4. In Vitro Cell Viability Test

**Figure 6:**
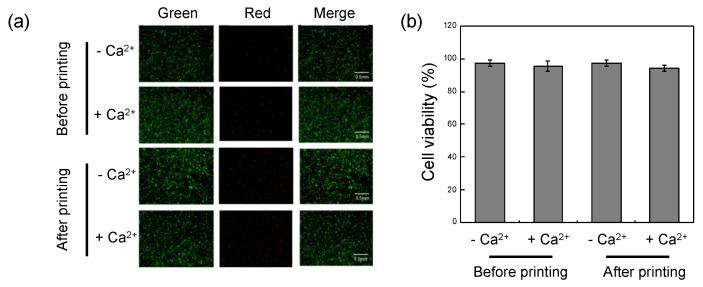
(**a**) Images of live and dead cells encapsulated within OHA/GC/ADH/ALG hydrogels before and after printing process. Secondary cross-linking of the gels was carried out with calcium ions (+Ca^2+^), and non-cross-linked gels were also used (−Ca^2+^). (**b**) Quantitative results from live/dead assays.

The cytotoxicity of each component of the hydrogel was evaluated using ATDC5 cells. OHA, GC, ADH, and ALG showed more than 95% cell viability even for a high level of material content, indicating that each hydrogel component has low cytotoxicity (Figure S2). A live/dead assay was conducted to determine whether cell viability was influenced by the methods of hydrogel fabrication, cross-linking, and printing. The shear force present during the printing process did not significantly influence cell viability. Furthermore, the second cross-linking process had little effect on cell viability (Figure 6). Therefore, this self-healable bioink system may be utilized for the fabrication of 3D scaffolds for the delivery of cells, drugs, and soluble factors in tissue engineering approaches.

### 3.5. Chondrogenic Differentiation In Vitro

**Figure 7:**
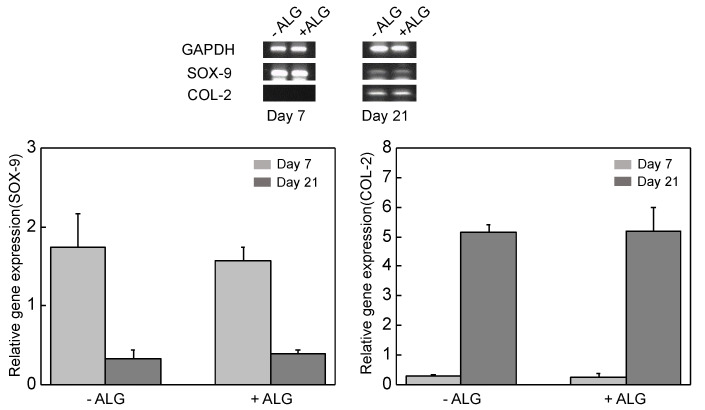
Quantification of gene expression level of ATDC5 cells cultured in vitro. The cells were encapsulated in OHA/GC/ADH gels (−ALG) or OHA/GC/ADH/ALG gels (+ALG) ([OHA] = 2 wt%, [GC] = 1 wt%, [ADH] = 0.3 wt%, [ALG] = 0.3 wt%, [Ca^2+^] = 60 mM).

OHA/GC/ADH/ALG hydrogel disks were formed by encapsulating the ATDC5 cells (1 × 10^7^ cells/mL). The disks were cultured in a bioreactor to provide an environment similar to that of the original tissue for 3 weeks. ATDC5 cells in the OHA/GC/ADH/ALG hydrogel maintained high expression level of early gene (*SOX-9*) at day 7 and high expression level of late gene (*COL-2*) at day 21 compared with the OHA/GC/ADH hydrogel (Figure 7). This result indicates that secondary cross-linking of OHA/GC/ADH/ALG hydrogels does not substantially influence the chondrogenic gene expression of ATDC5 cells in vitro.

The gel stiffness is known to be critical in the control of cell phenotype as well as cellular aggregation and chondrogenesis [37,38,39]. The mechanical stiffness of OHA/GC/ADH/ALG hydrogel increased after secondary cross-linking (Table 1, Figure 2). However, the change in the gel stiffness was not significant because a small amount of calcium ions was used. Interestingly, secondary cross-linking was useful to enhance the stability of the gels for 3 weeks (Figure 3), which may provide an environment suitable for the chondrogenic differentiation of ATDC5 cells.

## 4. Conclusions

A self-healing hydrogel, based on HA as a base biomaterial, was successfully fabricated with the simple mixture of GC and ADH. This hydrogel system was utilized as a bioink for 3D printing, owing to its self-healing abilities. In addition, secondary cross-linking after the printing process using ALG and calcium ions notably enhanced the mechanical stiffness and structural integrity of the gel. The amount of ALG and degree of ionic cross-linking were optimized for 3D printing based on rheological measurements of the gel, including storage shear modulus and gelation time. Additionally, it was determined that each hydrogel component, the secondary cross-linking process, and the printing process had little effect on the viability of ATDC5 cells encapsulated in the gel. It was also demonstrated that OHA/GC/ADH/ALG hydrogels can provide a microenvironment suitable for chondrogenic differentiation of ATDC5 cells in vitro. This self-healing bioink system may have great potential in many biomedical applications, including tissue and organ regeneration, using a 3D printer.

## Figures and Tables

**Table 1:** Effect of Second Cross-Linking on Storage Shear Modulus and Gelation Time of OHA/GC/ADH/ALG Self-Healing Hydrogel.

[ALG] (wt%)	0.3	0.6	0.9	1.2
G’ (Pa)	671 ± 27	535 ± 57	318 ± 51	N/A
complete gelation time	10 min	>20 min	>1 h	>1 h
G’ after second cross-linking (Pa)	948 ± 57	712 ± 71	671 ± 48	531 ± 51

## Data Availability

Not applicable.

## Supplementary Materials

The followings are available online at https://www.mdpi.com/article/10.3390/biomedicines9091224/s1, Figure S1: FT-IR spectra, Figure S2: Relative cell viability.
