# Supplementary material for: 3D Printing of Polysaccharide-Based Self-Healing Hydrogel Reinforced with Alginate for Secondary Cross-Linking
Source: Biomedicines. 2021 Sep 15;9(9):1224. doi: 10.3390/biomedicines9091224 (PMC8471923; doi:10.3390/biomedicines9091224)
Supplement: Supplementary file 1 [file biomedicines-09-01224-s001.zip › biomedicines-1298252-supplementary.pdf]

Supplementary Materials

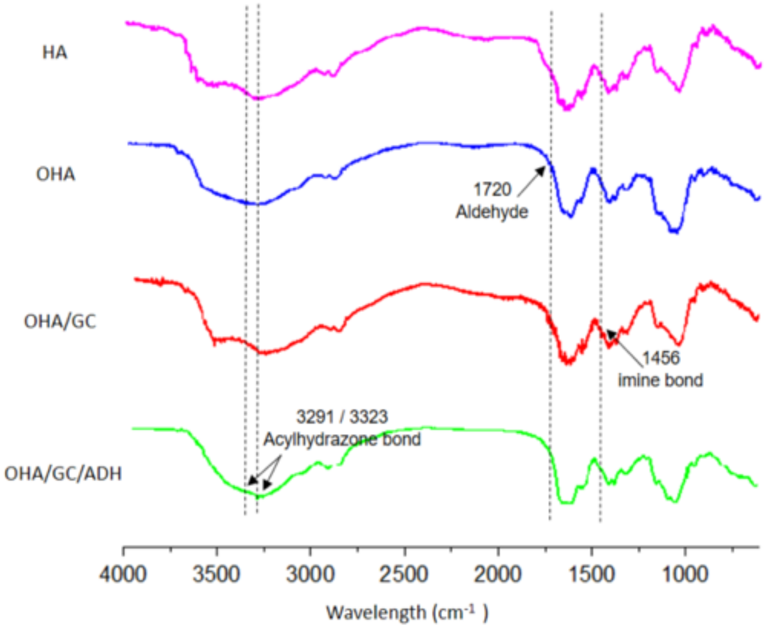

Figure S1. FT-IR spectra of HA, OHA, OHA/GC gel, and OHA/GC/ADH gel.

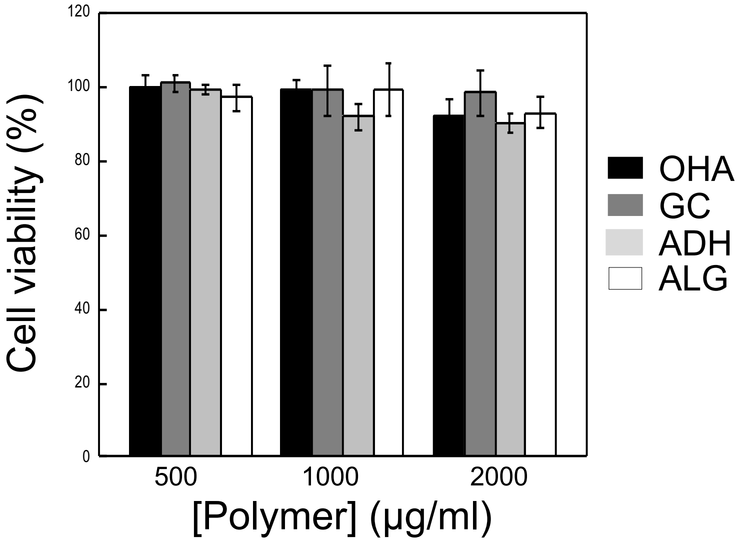

Figure S2. Relative viability of ATDC5 cells treated with each hydrogel component.
